# The complete chloroplast genome of *Quercus robur* ‘Fastigiata’

**DOI:** 10.1080/23802359.2019.1692724

**Published:** 2019-12-11

**Authors:** Lijuan Feng, Xuemei Yang, Qiqing Jiao, Chuanzeng Wang, Yanlei Yin

**Affiliations:** a Shandong Institute of Pomology, Taian, Shandong, China;; b Shandong Academy of Agricultural Sciences, Jinan, Shandong, China

**Keywords:** *Quercus robur* ‘Fastigiata’, chloroplast genome, Illumina sequencing, phylogenetic analysis

## Abstract

The *Quercus robur* ‘Fastigiata’ is an important ornamental plant, in which the complete chloroplast genome (accession no. MN562095) was identified and sequenced. The genome size is 161,172 bp, with a large single-copy (LSC, 90,505 bp) region, a small single-copy (SSC, 18,997 bp) region, and two inverted repeat regions (IRs, 25,835 bp each). A total of 134 genes are successfully annotated, including 89 protein-coding genes, 37 tRNA genes, and 8 rRNA genes. The phylogenetic relationships inferred that *Q. robur* ‘Fastigiata’ is closely related to *Quercus mongolica*, *Quercus wutaishanica*, and *Quercus dentata*.

The *Quercus robur* ‘Fastigiata’ is a very suitable plant for botanical gardens and parks, this is mainly distributed in France, Italy, USA, and China. It has high ornamental value and is suitable for planting in botanical gardens and parks (Iglesias-Díaz et al. 2000). The complete chloroplast (cp) genome can provide valuable genomic information for the conservation and restoration of rarely relict species (Qiang et al. 2019). The cp genome resources have been explored in *Quercus acutissima* (GenBank accession no. MH607377) (Li et al. 2018), *Quercus baronii* (GenBank accession no. KT963087) (Yang et al. 2015), and *Quercus dentata* (GenBank accession no. MG967555) (Hu et al. 2019), but the *Q. robur* ‘Fastigiata’ has not been fully sequenced. In this study, we sequenced and analyzed the cp genome of Fastigiata based on Illumina pair-end sequencing and compared it with other genus cp genome sequences. It is helpful for future genetic studies on this and other related species.

The voucher specimen (accession no. SDZXL00136) of *Q. robur* ‘Fastigiata’ tree was deposited in the Taidong field of Shandong Institute of Pomology, Shandong Province, China (36.20°N, 117.12°E). Total genomic DNA was extracted from young leaf tissue using the DNeasy Plant Mini Kit (Qiagen, Venlo, Netherlands). DNA was sequenced using the Illumina HiSeq 2500 platform by Genepioneer Biotechnologies (Nanjing, China). The raw paired-end reads were filtered using the fastp program (Chen et al. 2018). The high-quality reads were applied to a *de novo* assembly performed using GetOrganelle (Jin et al. 2018). Annotation was completed by the online program GeSeq (Tillich et al. 2017) and the result was manually adjusted where necessary using Geneious (Kearse et al. 2012). The complete cp genome was deposited in the GenBank (accession no. MN562095).

The complete cp genome of *Q. robur* ‘Fastigiata’ was 161,172 bp in length having 36.83% of total GC content. It is made up of a large single-copy (LSC, 90,505 bp) region, a small single-copy (SSC, 18,997 bp) region, and two inverted repeat regions (IRs, 25,835 bp each). A total of 134 genes are successfully annotated, including 89 protein-coding genes, 37 tRNA genes, and 8 rRNA genes. The content of protein-coding genes, tRNA genes, and rRNA genes is 66.4, 27.6, and 6.0%, respectively. The tRNA genes are distributed throughout the whole genome with 21 in the LSC, three in the SSC, and 13 in the IR regions, while rRNAs are only situated in the IR regions. Seven genes of tRNA (*trnA-UGC*, *trnI-CAU*, *trnI-GAU*, *trnL-CAA*, *trnN-GUU*, *trnR-ACG*, and *trnV-GAC*) had two copies and all four rRNA species (*rrn4.5*, *rrn5*, *rrn16*, and *rrn23*) also had two copies. Among the protein-coding genes, three genes (*clpP*, *rps12*, and *ycf3*) contained two introns, and other nine genes (*atpF*, *ndhA*, *ndhB*, *petB*, *petD*, *rpl16*, *rpl2*, *rpoC1*, and *rps16*) had one intron each.

The complete cp genome sequences of other Quercus, Trigonobalanus, Castanea, Fagus, Lithocarpus, and Castanopsis species were used to construct the phylogenetic tree, and *Acer catalpifolium* (GenBank accession no. MF179637) and *Acer wilsonii* (GenBank accession no. MG012225) as the outgroup. Using MAFFT v7.3 (Katoh and Standley 2013), we aligned 22 cp genomes of species. The maximum-likelihood (ML) phylogenetic tree was constructed by the IQ-TREE with the best-fit model identified using ModelFinder (Kalyaanamoorthy et al. 2017). The result showed that *Q. robur* ‘Fastigiata’ is closely related to *Quercus mongolica* (GenBank accession no. MK564083), *Quercus wutaishanica* (GenBank accession no. MK059753), and *Q. dentata* (GenBank accession no. MG967555) (Figure 1). This newly reported cp genome will provide valuable information for genetic evolution and molecular breeding studies of *Quercus*.

**Figure 1.**
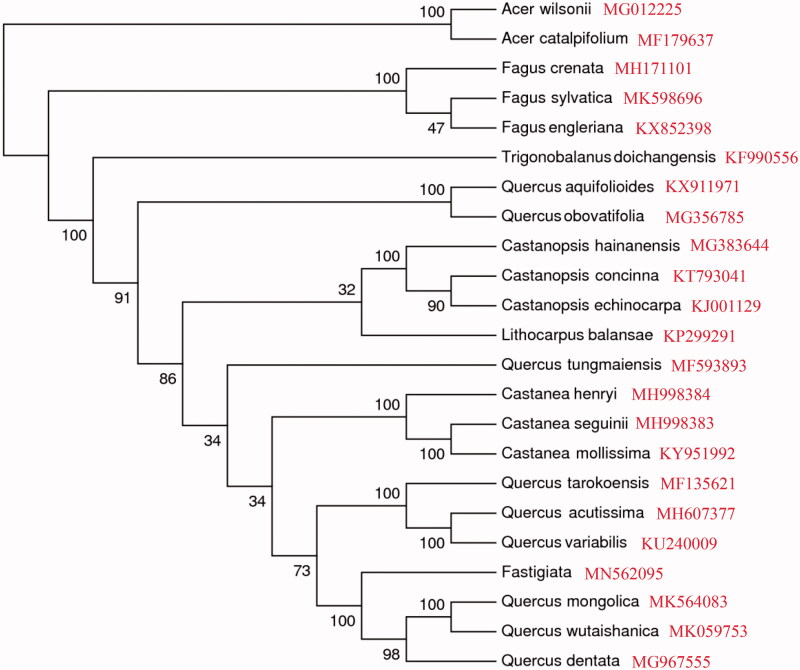
The phylogenetic tree of 22 complete chloroplast genome sequences based on the best maximum-likelihood (ML).
